# Differences in the Population Structure of Invasive *Streptococcus suis* Strains Isolated from Pigs and from Humans in the Netherlands

**DOI:** 10.1371/journal.pone.0033854

**Published:** 2012-05-01

**Authors:** Constance Schultsz, Ewout Jansen, Wendy Keijzers, Anja Rothkamp, Birgitta Duim, Jaap A. Wagenaar, Arie van der Ende

**Affiliations:** 1 Academic Medical Center, Department of Global Health, University of Amsterdam, Amsterdam, The Netherlands; 2 Oxford University Clinical Research Unit, Hospital for Tropical Diseases, Ho Chi Minh City, Vietnam; 3 Center for Infection and Immunity Amsterdam (CINIMA), Department of Medical Microbiology, Academic Medical Center, Amsterdam, The Netherlands; 4 The Netherlands Reference Center for Bacterial Meningitis, Academic Medical Center, Amsterdam, The Netherlands; 5 GD - Animal Health Service, Deventer, The Netherlands; 6 Department of Infectious Diseases and Immunology, Faculty of Veterinary Medicine, Utrecht University, Utrecht, The Netherlands; University of Iowa, United States of America

## Abstract

*Streptococcus suis* serotype 2 is the main cause of zoonotic *S. suis* infection despite the fact that other serotypes are frequently isolated from diseased pigs. Studies comparing concurrent invasive human and pig isolates from a single geographical location are lacking. We compared the population structures of invasive *S. suis* strains isolated between 1986 and 2008 from human patients (N = 24) and from pigs with invasive disease (N = 124) in the Netherlands by serotyping and multi locus sequence typing (MLST). Fifty-six percent of pig isolates were of serotype 9 belonging to 15 clonal complexes (CCs) or singleton sequence types (ST). In contrast, all human isolates were of serotype 2 and belonged to two non-overlapping clonal complexes CC1 (58%) and CC20 (42%). The proportion of serotype 2 isolates among *S. suis* strains isolated from humans was significantly higher than among strains isolated from pigs (24/24 vs. 29/124; P<0.0001). This difference remained significant when only strains within CC1 and CC20 were considered (24/24 vs. 27/37,P = 0.004). The Simpson diversity index of the *S. suis* population isolated from humans (0.598) was smaller than of the population isolated from pigs (0.765, P = 0.05) indicating that the *S. suis* population isolated from infected pigs was more diverse than the *S. suis* population isolated from human patients. *S. suis* serotype 2 strains of CC20 were all negative in a PCR for detection of genes encoding extracellular protein factor (EF) variants. These data indicate that the polysaccharide capsule is an important correlate of human *S. suis* infection, irrespective of the ST and EF encoding gene type of *S. suis* strains.

## Introduction

*Streptococcus suis* is an emerging human pathogen. The number of human *S. suis* infections reported worldwide has increased significantly in the past years, with most cases originating in Southeast Asia [1]. Human *S. suis* infection is acquired through exposure to contaminated pigs or pig derived products. Meningitis and sepsis are the most common clinical manifestations of *S. suis* infections; hearing loss is a frequent complication [1].

*S. suis* infections are causing huge losses in pig production worldwide. Although multiple putative virulence factors of *S. suis* have been identified [2], [3], [4], [5], including the polysaccharide capsule [6], [7], none of these have been shown to be essential for infection. The *epf* gene encoding Extracellular Protein Factor (EF), is a virulence-associated gene and is used as a virulence marker although an *epf* mutant was not severely attenuated in virulence in experimental infection [8], [9]. Repeats-containing variants of this gene (epf*, encoding EF*) were found in non-pathogenic or weakly pathogenic strains [10]. In addition, Muramidase Released Protein (MRP) and the hemolysin (suilysin), have been used as virulence markers [8], [11].

Healthy pigs can carry multiple serotypes of *S. suis* in their nasal cavities, tonsils, and upper respiratory, genital and alimentary tracts. Of the 33 serotypes, serotypes 1–9 and 14 are most commonly associated with clinical infections in pigs [12], [13]. Across the infectious serotypes, differences in virulence can be observed under experimental conditions. Thus, serotype 2 is considered to be more pathogenic for pigs than serotype 9 [14]. However, the latter serotype is endemic in the pig population causing clinical illness under conditions such as stress and suboptimal farm management, in certain geographical areas [12]. *S. suis* serotype 2 is the most common cause of *S. suis* infection in humans, in contrast to the situation in pigs. Serotypes 1, 4, 14 and 16 have been reported in a limited number of patients only [1]. It is unknown why isolates with serotypes which are highly prevalent amongst diseased pigs, such as serotypes 7 and 9, have not been isolated from human cases of *S. suis* infections. One possible explanation for this observation is the lack of human exposure to strains with serotypes 7 and 9 due to a much higher prevalence of serotype 2 strains in pigs in geographical areas where humans are highly exposed. However, another plausible explanation is that humans are more susceptible to infection with serotype 2 strains than to infection with strains of serotypes 7 or 9.

Multi Locus Sequence Typing (MLST) is a recognized tool for characterization of bacterial population structures. A MLST scheme was designed for *S. suis* [15], and the currently available comprehensive data set indicates that strains belonging to clonal complex 1 (CC1), which includes sequence type 1 (ST1), are associated with invasive disease in both pigs and humans. Other CCs including invasive strains isolated from both humans and pigs include CC25, CC28, and CC104 [16], [17].

*S. suis* infection is a well recognized problem within the pork-rearing industry in the Netherlands [18]. In addition, human cases of *S. suis* infection occur regularly in the Netherlands albeit at a very low frequency [19], [20], [21]. Surveillance of *S. suis* infections and storage of bacterial isolates for both animals and humans are centralized. The combined sets of stored invasive isolates provide a unique opportunity to study and compare the population structures of *S. suis* strains isolated from humans and pigs with invasive disease in a single geographical area in order to identify differences across *S. suis* strains infecting humans and pigs.

## Materials and Methods

### Bacterial Strains

All isolates cultured from cerebrospinal fluid or blood from patients with bacterial meningitis in the Netherlands are submitted to the National Reference Laboratory of Bacterial Meningitis (NRLBM) at the Academic Medical Centre, University of Amsterdam, for further typing and storage, as part of the continuous surveillance of bacterial meningitis in the Netherlands. For this study the isolates were anonymized. Additional institutional review board approval is not required for studying anonymized submitted strains without patients data.

All *S. suis* strains, isolated from cerebrospinal fluid or blood of human patients between 1986 and 2007, which were available at the NRLBM, were included in the study.

*S. suis* isolates from pigs were obtained from samples sent to the Animal Health Service, Deventer, or the Veterinary Microbiological Diagnostic Center of the Faculty of Veterinary Medicine, Utrecht in the Netherlands. These samples were submitted by veterinary practitioners for diagnosis of *S. suis* infection. Anonymized non-duplicate invasive *S. suis* isolates isolated in the period 1996–2008, were used in this study. A representative sample of each year from a total number of 2773 strains was obtained by choosing every 20^th^ isolate irrespective of source or serotype. *S. suis* isolates obtained from pigs were defined as invasive if they were cultured from brain tissue, cerebrospinal fluid, blood, or joints from pigs with clinical disease compatible with *S. suis* infection.

Strains were identified using Gram-stain, catalase test and API20Strep (bioMerieux). Serotyping was performed using PCR for detection of serotypes 1 (and 14), 1/2 and 2, 7, and 9 as described by Wisselink and colleagues [22], including positive and negative control strains, and by slide agglutination using serotype specific antibodies for strains which were untypeable by PCR (H. Smith, Central Veterinary Institute, Lelystad, The Netherlands).

### Multi Locus Sequence Typing

Multi Locus Sequence Typing (MLST) was performed as described by King et al. [15]. The nucleotide sequences of 7 housekeeping genes were generated by PCR and sequencing, using primers as described in the *S. suis* MLST scheme, and the sequence type (ST) was determined on the basis of the *S. suis* MLST database available at www.mlst.net. To identify clonal complexes, i.e groups of related genotypes (STs), isolates were grouped with all isolates present in the *S. suis* database using the eBURST algorithm (http://eburst.mlst.net) with the software provided by the MLST website. Clonal complexes consisted of sequence types that shared 6 of 7 alleles with at least 1 other sequence type in the complex and named after the putative founder (i.e. the ST that has the greatest number of single-locus variants) of the group or after the most frequent ST of the group. Sequence types that did not group with other sequence types in the database were defined as singletons. For calculation of the genetic diversity amongst isolates cultured from humans or from pigs, the Simpson’s index of diversity was used, 
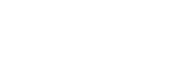
 where n_i_ is the number of strains belonging to i^th^ type and N is the total number of strains in the sample population [23].

### Phylogenetic Analyses

A distance matrix in Nexus format was generated from the set of allelic profiles using SplitsTree at http://pubmlst.org/analysis/. This file was then used for phylogenetic analyses in SplitsTree 4.0, by generating a Neighbor-Joining tree [24].

### *epf* gene Typing

The presence of the *epf* gene, and its larger size variants designated epf* encoding EF or EF*, was determined for all strains isolated from human patients using PCR, as described by Wisselink et al. [22].

## Results

### Human Isolates

During the period 1986–2007, 24 *S. suis* isolates obtained from human patients with meningitis were submitted to the NRLBM. Submitted isolates were equally distributed over time and originated from multiple provinces with a high pig density, throughout the country. All patients were male and were aged between 27 and 66 years. Exposure to pigs or pork was confirmed for 15 (63%) of patients, 14 of whom were keeping pigs or were working in slaughterhouses.

All 24 human strains were identified as serotype 2 strains using PCR, with a PCR product of the expected size for serotype 2 and a negative PCR for detection of serotype 1 (Table 1 and supplementary Table S1). MLST identified two distinct populations. One group of 12 strains (50%) was assigned to ST1 (CC1) whilst the other group of 10 strains (42%) to ST20 (CC20). Two strains were assigned new sequence types (ST134, ST146) on the basis of sequence variation within a single locus when compared with allele sequences of ST1 (single locus variants). Strains of ST1 and ST20 were equally distributed over time without clustering.

**Table 1:** Distribution of *Streptococcus suis* isolates among patients and pigs in the Netherlands, according to serotype and genotype.

		Genotype (Clonal Complex)							
host	serotype	1	13	16	20	25	27	Singleton	total
human	2	14			10				24
									
pig	1	3	4						7
	1/2	1					1		2
	2	24			3	1			28
	3			2					2
	4				3				3
	7			1		5		3	9
	8			2					2
	9	2		62	1			5	70
	NT							1	1
All		44	4	67	17	6	1	9	148

All strains with ST1 or single locus variants of ST1 were positive in the PCR for detection of the *epf* gene. Ten strains carried the *epf* gene and 4 strains the epf* gene. All strains with ST20 were negative in this PCR.

### Pig Isolates

A total of 130 pig isolates were obtained from the collection. Six strains did not yield the expected PCR products in the MLST assays and were excluded from further analysis. *S. suis* strains isolated from pigs had a much more diverse serotype distribution than strains isolated from humans (Table 1 and Table S1). Serotype 9 was the predominant serotype. Amongst 124 pig isolates, 70 (56%) had serotype 9 and 29 (23%) serotype 2 (p<0.001, χ2 test; human vs pig strains for serotype 2).

Sequence typing showed more diversity for pig isolates than for human isolates. Of 124 strains typed by MLST, 53 (43%) had ST16 and 28 (23%) had ST1. ST20 was found in only 3 strains isolated from pigs (p<0.001, Fisher’s exact test; human vs pig strains for ST20). A new ST was defined for each of 19 pig isolates (Table S1). The higher number of genotypes (STs) and CCs among *S. suis* strains isolated from pigs indicated that the *S. suis* population isolated from infected pigs was more diverse than the *S. suis* population isolated from human patients. Comparison of diversity of the *S. suis* populations isolated from pigs and humans using the Simpson’s diversity index, showed a significant difference for both STs (0.775 and 0.598, respectively, P = 0.05; T-test) and CCs (0.648 and 0.507, respectively; P = 0.05; T-test).

### Association between Genotype and Serotype

**Figure 1:**
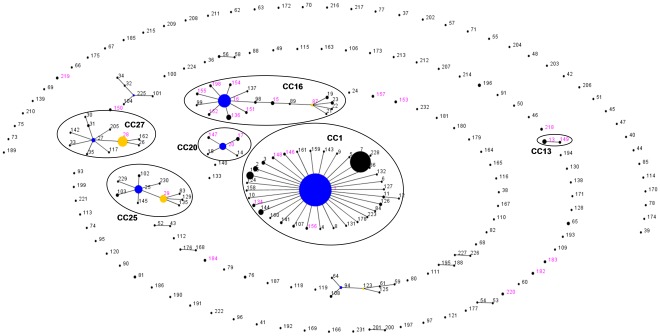
Combined eBURST analysis of the entire *S. suis* MLST database (accessed on 2 August 2011) and the complete collection of strains isolated from pigs and humans with invasive *S. suis* infection described in this study. Clonal complexes and the predicted founder STs are indicated by blue dots. Secondary founders are indicated by yellow dots. The size of the dots is relative to the number of isolates with the respective ST present in the combined databases. Numbers in magenta correspond to the STs of the Dutch isolates in this study. Clonal complexes relevant to this study are circled and labeled. Isolates cultured from human patients in this study are of CC1 (ST1, ST134 and ST148); and of CC20 (ST20).

To assess the clonal complex distribution among the Dutch isolates, isolates were grouped with all isolates (823 isolates on August 2, 2011) present in the MLST database at http://ssuis.mlst.net/, using ST profiles in an eBURST analysis (Figure 1). Among 148 isolates, comprising 30 unique STs, 6 clonal complexes were identified. CC1, CC13, CC16, CC20, CC25 and CC27 comprised 44, 4, 67, 17, 6 and 1 isolates, respectively (Table 1, Table S1). The remaining 9 isolates were singletons. Of 124 pig isolates, 67 (54%) were of CC16. Human isolates were only seen in CC1 and CC20. Among human isolates the proportion of isolates belonging to CC1 (58%) or to CC20 (42%) was significantly higher than among pig isolates, (24%, p<0.002, χ2 test; and 6% (p<0.0001, Fisher exact test; respectively).

The distribution of clonal complexes across the different serotypes is shown in Table 1 and supplementary Table S1. Of 44 CC1 isolates, 38 (86%) were of serotype 2, 3 of serotype 1, 1 of serotype 1/2 and 2 of serotype 9. Of 17 CC20 isolates, 13 (76%) were of serotype 2, 3 of serotype 4, and one of serotype 9. The vast majority of CC16 isolates (all from pigs) were of serotype 9 (62/67; 93%), 2 were serotype 3, one serotype 7 and two serotype 8.

Thus the two major serotypes 2 and 9 were distributed over multiple generally non-overlapping clonal complexes. Serotype 2 strains were found in CC1 (38/53; 72%), CC20 (13/53; 25%), CC27 (1/53; 2%) and CC29 (1/53; 2%). Serotype 9 comprised isolates of CC1 (2/70; 3%), CC16 (62/70; 89%), and CC20 (1/70; 1%), as well as 5 (7%) singletons.

The proportion of serotype 2 strains among *S. suis* strains isolated from humans was significantly higher than among strains from pigs (24/24 vs 29/124; P<0.001). This difference remained significant when only strains belonging to CC1 and CC20 were considered. Amongst CC1 and CC20 strains, all 24 strains (100%) isolated from humans were of serotype 2, compared with 27 of 37 (73%) pig strains (P = 0.004; Fisher’s exact).

### Phylogenetic Analysis

**Figure 2:**
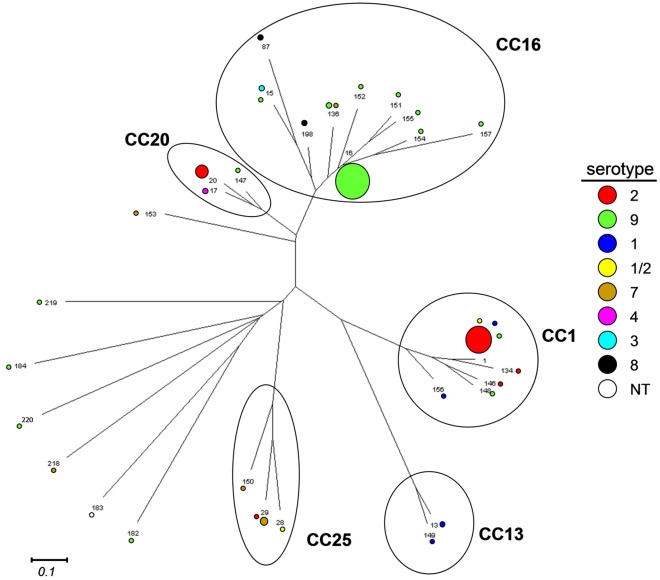
Phylogenetic analyses of concatenated sequences of seven housekeeping gene fragments of *Streptococcus suis* strains isolated from human patients and pigs in the Netherlands. The tree was constructed using Neighbor-Joining algorithm in SplitsTree4 using MLST allelic profiles. Distance matrix was obtained from allelic profiles using the SplitsTree program at http://pubmlst.org/analysis/. ST’s comprising the different clonal complexes are circled. Serotypes are indicated by coloured dots with a diameter corresponding to the number of strains. The horizontal line indicates the scale for genetic distance in arbitrary units.

A Neighbor-Joining cluster analysis of allelic profiles using SplitsTree4 showed groups corresponding with clonal complexes identified with eBURST (Figure 2). Again, clusters comprised multiple STs as well as serotypes. Moreover, some STs were associated with multiple serotypes.

## Discussion

We observed significant differences between the *S. suis* populations isolated from human patients and from diseased pigs in the Netherlands. Whilst serotype 9 was predominant amongst invasive pig isolates, serotype 2 was responsible for all human infections, during the same time period. In case series and other reports on *S. suis* infections in humans, serotype 2 strains were responsible for more than 95% of infections [1]. Limited data are available on the prevalence of serotype 2 and other serotypes in the pig populations in areas where most human infections occur, such as in Vietnam and Thailand. A high prevalence of invasive serotype 2 strains in the pig population may explain a predominance of these strains as a cause of zoonotic infections. Indeed, in a study on the characteristics of invasive strains isolated from diseased pigs in China, the most prevalent serotype was serotype 2, followed by serotype 3 [25], supporting this view. In addition, serotype 2 was the most prevalent serotype carried in the tonsils of healthy slaughterhouse pigs in Vietnam [26]. In contrast, serotype 9 was the most prevalent serotype in diseased pigs in our study performed in the Netherlands, but this serotype was not isolated from human patients. Data on the prevalence of serotype 2 and serotype 9 strains in healthy pigs in the Netherlands are not available. However, since serotype 2 is considered more virulent than serotype 9 [14], one would expect this serotype to be isolated more frequently from diseased pigs than serotype 9 strains if serotype 2 was more prevalent than serotype 9 in the healthy pig population.

The number of human strains studied is relatively small compared with the number of pig strains. In addition, invasive pig strains were isolated in a period only partly spanning the period in which human strains were isolated (1996 to 2007 vs 1986 to 2007). We analyzed the distribution of genotypes of the human isolates in time and found similar distributions in the two periods 1986 to 1995 (6 strains each CC1 and CC 20) and 1996 to 2007 (8 strains CC1, 4 strains CC20, Fisher’s exact test not significant). The population of invasive pig *S. suis* strains was more diverse than that of *S. suis* isolated from human patients as indicated by the estimated Simpson’s diversity indices, suggesting selection in the transmission from pigs to human. Human patients in the Netherlands appear to be infected by only two, distinct, clonal complexes of *S. suis*. Whilst each of these clonal complexes is associated with multiple serotypes, human patients were infected only by *S. suis* serotype 2. The structure of the serotype 2 capsular polysaccharide of *S. suis* was recently determined. The backbone sequence was found to be identical to that of *Streptococcus agalactiae* type VIII and *Streptococcus pneumoniae type* 23F, whilst the repeating unit contained a terminal 2,6-linked sialic acid molecule. Bacterial capsular sialic acid has been shown to contribute to immune evasion in a number of pathogens [27], [28]. Although reports on the possible role of sialic acid in porcine disease pathogenesis during *S. suis* infection were inconclusive [29], [30], it is tempting to speculate on the role of capsular sialic acid in human *S. suis* infection. Strikingly, the only other serotype of *S. suis* increasingly found to infect humans, albeit at low numbers, is serotype 14 [31], [32], [33], which was shown to also contain genes involved in sialic acid synthesis, in contrast to serotype 7 and 9 strains [34]. Serotype 14 strains were not observed in our study. Although the capsule of serotype 1 also contains sialic acid [34], human infection with *S. suis* of serotype 1 has only been reported once [35]. In our study, 7/124 (6%) of the porcine isolates were of serotype 1 making transfer from pigs to human less likely than that of serotype 2 isolates (29/124 [23%]).

EF, encoded by the *epf* gene, is associated with but not essential for virulence. Similarly, other proteins, such as Muramidase Released Protein (MRP) and the hemolysin (sly) have been associated with virulence and these proteins are now often used as markers to differentiate virulent and less-virulent strains. Amongst the serotype 2 strains which were isolated from human patients, those with ST20 (CC20) were negative in a PCR for detection of the *epf* gene or its high molecular weight variants, as opposed to the strain belonging to CC1. This indicates that EF is not required for *S. suis* serotype 2 invasive disease caused by ST20 strains in human patients and suggests that the presence of these genes is associated with the genotype.

Serotype 2 strains which were isolated from human patients were of ST1, ST134 or ST146 (CC1), and of ST20 (CC20). Whilst ST1 is known to contain virulent strains which occur worldwide, invasive isolates with ST20 have not been described before. One isolate of serotype 9 (ST147, CC20) from a diseased pig appeared to be a single locus variant of ST20. These findings, in addition to the observation that multiple serotypes are found in CC16 and CC1, indicate that capsule switch due to horizontal transfer of capsule loci occurs rather frequently in *S. suis*, as was described earlier by King and colleagues [15]. The high prevalence of ST20/CC20 strains amongst the *S. suis* strains isolated from humans in our study, as well as the recently reported strains of clonal complex ST104 isolated from human patients in Thailand, indicate that the population of *S. suis* serotype 2 strains infecting humans is more diverse than previously suspected. Enhanced surveillance of *S. suis* infections in humans may further increase our knowledge on the population of *S. suis* causing zoonotic infection.

Fifty-four percent of the *S. suis* isolates from pigs belonged to CC16, while the remaining 42% were distributed over five clonal complexes and 9 singletons. Serotypes within CC16 included serotypes 9, 7, and 8. These data indicate that CC16 is another clonal complex representing strains with invasive potential in pigs, in addition to CC1, CC28 and CC25. The high prevalence of ST16 strains in our study, which originated from different farms, regions and were isolated in different years, resulted in ST16 becoming the founder of the CC16 (CC87) complex.

In summary, in this study from the Netherlands, humans were infected by *S. suis* serotype 2 only, while serotype 9 was most prevalent among diseased pigs. Human patients were infected by two distinct genotypes or clonal complexes (CC1 and CC20). In contrast, strains belonging to CC16 were most frequently isolated from infected pigs. Within CC1 and CC20, serotype 2 was strongly associated with disease in humans. These data indicate that the polysaccharide capsule is an important correlate of human *S. suis* infection, irrespective of the ST and EF encoding gene type of *S. suis* strains.

## Supporting Information

Table S1 Characteristics of *Streptococcus suis* strains included in the study.
(XLS)
